# Recent advances in nutritional composition, phytochemistry, bioactive, and potential applications of *Syzygium aromaticum* L. (Myrtaceae)

**DOI:** 10.3389/fnut.2022.1002147

**Published:** 2022-10-14

**Authors:** Qing Xue, Zedong Xiang, Shengguang Wang, Zhufeng Cong, Peng Gao, Xiaonan Liu

**Affiliations:** ^1^College of Pharmaceutical Science, Shandong University of Traditional Chinese Medicine, Jinan, Shandong, China; ^2^Shandong Provincial Institute of Cancer Prevention and Treatmen, Jinan, Shandong, China; ^3^Chinese Medicine Innovation Research Institute, Shandong University of Traditional Chinese Medicine, Jinan, Shandong, China

**Keywords:** *Syzygium aromaticum*, medicinal food, nutritional composition, phytochemistry, bioactivities, applications

## Abstract

*Syzygium aromaticum* is an aromatic plant native to Indonesia, and introduced to tropical regions worldwide. As an ingredient in perfumes, lotions, and food preservation, it is widely used in the food and cosmetic industries. Also, it is used to treat toothache, ulcers, type 2 diabetes, etc. A variety of nutrients such as amino acids, proteins, fatty acids, and vitamins are found in *S. aromaticum*. In addition to eugenol, isoeugenol, eugenol acetate, β-caryophyllene and α-humulene are the main chemical constituents. The chemical constituents of *S. aromaticum* exhibit a wide range of bioactivities, such as antioxidant, antitumor, hypoglycemic, immunomodulatory, analgesic, neuroprotective, anti-obesity, antiulcer, etc. This review aims to comprehend the information on its taxonomy and botany, nutritional composition, chemical composition, bioactivities and their mechanisms, toxicity, and potential applications. This review will be a comprehensive scientific resource for those interested in pursuing further research to explore its value in food.

## Introduction

Spices are considered to be one of the earliest recorded functional foods, with international trade in spices dating back as far as 4500–1900 B.C. They are usually aromatic, dried plant parts obtained from seeds, fruits, leaves, roots, and bark (1). More than 100 species of plants are currently used worldwide as spices and flavorings that play an important role in cooking, health care, and preserving (2). In addition, spices serve as a rich reservoir of bioactive compounds and also have antioxidant, antibacterial, anti-inflammatory, antidiabetic, and anticancer properties that help fight various diseases in the human body (3).

**FIGURE 1:**
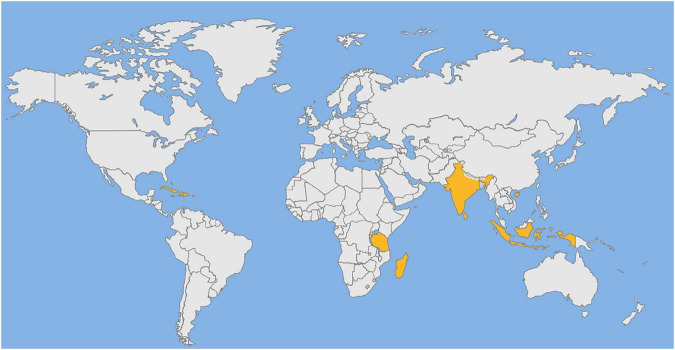
Distribution of *S. aromaticum* around the world. West Indies, Tanzania, Madagascar, India, Sri Lanka, Indonesia, Malaysia and Hainan province of China.

*Syzygium aromaticum* L. (Myrtaceae), is also known as *Eugenia caryophyllata Thunb.* It is an evergreen tree native to the Maluku Islands in Indonesia (4). Its dried flower buds are referred to as “clove,” it is highly sought-after for medicinal and culinary purposes. In medieval Europe, it is cultivated for commercial purposes. Currently, it is produced mainly in Indonesia, West Indies, Madagascar, India, Tanzania, and Sri Lanka (5). especially Zanzibar and Pemba Island in Tanzania, which produce about 80% of the world’s production (6) (Figure 1). Clove has been used as a spice in ancient China for more than 2000 years and was included in the first list of medicinal food items in China in 2002. Recent studies have found that fruits, seeds and leaves of *S. aromaticum* also contain bioactive compounds. The Chinese Traditional Medicine Administration has listed the fruit as one of the 39 herbal species that should be given priority for further development. Despite having commercial and medicinal value, it is not been extensively reviewed on its nutritional composition and advances in its chemistry, pharmacology, toxicology, and applications. This review is the first time for the comprehensive analysis of the nutritional components of its various parts, which has never appeared in previous papers. Secondly, in terms of chemical composition, although some articles have introduced it, this paper summarizes the chemical compositions of each part for the first time and classifies them into volatile and non-volatile components. Thirdly, in terms of biological activity, although some articles introduced the biological activity of *S. aromaticum*, there was no detailed and clear mechanism diagram. In this paper, the biological activities of *S. aromaticum* were comprehensively summarized and the mechanism diagrams were drawn for the first time. Fourthly, in terms of application, most of the existing articles only introduced the fresh-keeping effect of clove. In this paper, the applications of *S. aromaticum* in medicine and food have been comprehensively analyzed for the first time, and the relevant popular contents of nanometer preparations have been added. Overall, this review will be a comprehensive scientific resource for those interested in pursuing further research to explore its value in medicine and food.

## Taxonomy and botanical description

*Syzygium aromaticum* belongs to the Myrtales order, Myrtaceae family, and Syzygium genus. Myrtaceae family plants are mainly grown in tropical and subtropical regions of Australia and America (7). Syzygium is an important genus in the Myrtaceae family and contains more than 500 species, mainly distributed in tropical Asia and a few species in Oceania and Africa (8). *S. aromaticum* belongs to the plant kingdom, Angiospermae, Dicotyledoneae, and Archichlamydeae.

**FIGURE 2:**
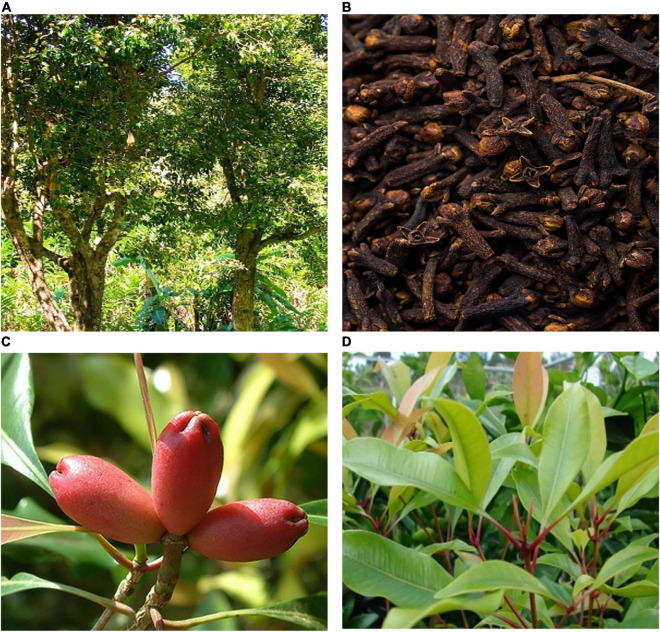
Different parts of *S. aromaticum*. **(A)** Whole plant; **(B)** dried flower buds; **(C)** fruit; **(D)** leaf. Pictures are from https://baike.baidu.com/pic/%E4%B8%81%E5%AD%90%E9%A6%99/19435258.

*Syzygium aromaticum* is an evergreen tree growing to about 10–20 m. The bark is gray and smooth. The leaves are large and opposite. The leaf blades are leathery, ovate-long elliptic, entire, and densely covered with oil glands. The petiole is conspicuous. The leaf buds are terminal, cymes or panicles. The flowers are red or pink, and 3 flowers are together in one group. The flowers contain 4 petals. The buds are white at the beginning and turn green and then red when the buds are 1.5 to 2cm long (9). The calyx is cylindrical, receptacle long, and 4-lobed at the tip. The lobes are triangular, bright red, with numerous stamens and an inferior ovary. The berries are oblong, red or dark purple, and contain one seed, which is oval. The fruit is called female or female *S. aromaticum*. The fruiting period is from June to July, and the flowering period is from January to February. The buds are called male or male *S. aromaticum*, and dried by removing the pedicel from the bright red buds (10). The different parts of *S. aromaticum* are shown in Figure 2.

## Nutritional composition

The flower buds, fruits, branches, leaves, and seeds of *S. aromaticum* are rich in various nutrients, proteins, fatty acids, mineral elements, amino acids, vitamin, etc. The presence of these nutrients makes *S. aromaticum* as a plant with high economic value. The nutritional composition of each part of the plant is listed in Tables 1–4. Only a very few studies were conducted to identify the vitamins present in it, and the vitamins present in it are listed in Table 5.

**TABLE 1:** Conventional nutrients in various parts of *S. aromaticum*.

Nutrient composition	Buds			Fruits	Branches	Leaves	Seeds
	(mg/g) (11)	(%) (12)	(mg/g) (13)	(mg/g) (11)	(mg/g) (11)	(mg/g) (11)	(%) (14)
Moisture	−	9.63 ± 0.19	68.6	−	−	−	7.74 ± 0.2
Total carbohydrate	86.75 ± 5.61	8.26 ± 0.16	612.1	168.12 ± 8.92	106.47 ± 7.23	88.13 ± 6.13	51.3 ± 2.7
Crude protein	39.8 ± 3.79	6.06 ± 0.12	59.8	33.73 ± 4.26	45.1 ± 3.38	61.75 ± 5.62	6.9 ± 0.4
Crude fiber	111.72 ± 9.73	9.64 ± 0.32	342	73.38 ± 7.35	368.55 ± 23.45	253.13 ± 21.12	11.47 ± 0.5
Crude fat	123.58 ± 11.3	59.3 ± 0.15	200.7	25.78 ± 1.73	10.35 ± 0.26	41.56 ± 4.23	16.63 ± 0.3
Ash	34.17 ± 1.03	5.36 ± 0.08	58.8	23.08 ± 1.34	21.91 ± 1.53	40.59 ± 2.86	5.96 ± 0.1

“–” indicates that the value is not available.

**TABLE 2:** Fatty acid composition in various parts of *S. aromaticum*.

Fatty acid	Buds	Fruits	Branches	Leaves		Seeds
	(%) (11)	(%) (11)	(%) (11)	(%) (11)	(%) (15)	(%) (14)
C12:1 (n-7)	–	–	–	0.54	–	–
C14:0	3.35	2.24	3.41	2.64	0.37	1.29
C15:0	–	–	–	0.29	–	–
C16:0	32.14	38.30	47.69	43.29	7.23	6.21
C16:1 (n-7)	–	0.66	–	0.28	–	20.96
C17:0	–	–	1.42	1.10	–	–
C18:0	13.42	18.55	21.14	12.74	5.08	–
C18:1 (n-7)	–	–	–	0.44	0.44	6.20
C18:1 (n-9)	8.72	14.81	6.26	12.23	16.12	13.0
C18:1 (n-12)	–	–	1.39	–	–	–
C18:2 (n-6)	33.72	21.28	13.81	15.93	36.58	44.73
C18:3 (n-3)	8.21	4.16	4.88	9.80	30.55	2.93
C20:1 (n-9)	–	–	–	0.55	–	–
C20:0	–	–	–	–	2.82	4.68
C22:0	–	–	–	–	1.25	–
SFA	48.91	59.09	73.66	60.06	16.75	12.18
MUFA	8.72	15.47	7.65	14.04	16.12	40.16
PUFA	41.93	25.44	18.69	25.73	67.13	47.66

“–” indicates that the value is not available; SFA, saturated fatty acid; MUFA, monounsaturated fatty acid; PUFA, polyunsaturated fatty acid.

**TABLE 3:** Mineral elements content in various parts of *S. aromaticum*.

Mineral elements	Buds			Fruits	Branches	Leaves	Seeds	
	(mg/kg) (11)	(mg/kg)(12)	(mg/kg) (13)	(mg/kg) (11)	(mg/kg) (11)	(mg/kg) (11)	(mg/100g)(14)	(μ g/g)(16)
Potassium (K)	14 087.39	14 784.916 7	11 020	8 540.36	3 509.68	9 315.80	650.0 ± 30.0	−
Calcium (Ca)	6 888.94	6 302.166 7	6 460	2 949.98	6 294.30	9 299.14	270.0 ± 20	−
Sodium (Na)	3 995.71	4 143.166 7	2 430	2 554.04	1 660.37	3 105.66	−	−
Magnesium (Mg)	3 406.92	3 036.549 0	2 640	1 402.07	751.32	1 884.82	97.0 ± 10.0	−
Manganese (Mn)	664.88	858.033 4	300.33	411.57	430.01	870.30	43.8 ± 1.5	736.36 ± 40.42
Iron (Fe)	544.48	1 292.349 0	86.8	1 364.26	61.47	61.41	36.0 ± 0.8	4.26 ± 0.15
Aluminum (Al)	161.58	759.783 3	-	427.40	31.54	45.89	−	4.21 ± 0.34
Phosphorus (P)	-	-	1050	-	-	-	−	−
Strontium (Sr)	62.16	40.859 3	-	14.90	26.58	23.26	−	4.74 ± 0.06
Boron (B)	49.29	29.536 7		12.37	12.79	36.09		
Barium (Ba)	11.27	17.730 9	-	5.12	8.93	8.83	−	3.57 ± 0.23
Thallium (Ti)	14.61	87.541 3	-	15.14	9.99	12.02	−	−
Zinc (Zn)	13.10	10.252 5	10.9	4.04	6.84	7.60	0.7 ± 0.1	5.97 ± 4.49
Copper (Cu)	2.65	4.004 3	3.47	1.87	1.20	1.21	0.8 ± 0.1	3.55 ± 0.22
Stannum (Sn)	0.42	0.018 1	-	0.15	0.14	0.15	−	−
Vanadium (V)	0.17	5.308 7	-	0.64	0.12	0.06	−	−
Chromium (Cr)	-	0.847 500	-	-	-	-	−	0.05 ± 0.01
Nickel (Ni)	-	0.578 333	-	-	-	-	−	4.25 ± 0.39
Arsenic (As)	-	0.018 833	-	-	-	-	−	0.05 ± 0.01
Plumbum (Pb)	-	0.100 083	-	-	-	-	−	−
Hydrargyrum (Hg)	-	0.019 6250	-	-	-	-	−	−
Molybdenum (Mo)	0.05	-	-	0.07	0.03	0.08	−	−
Selenium (Se)	0.02	-	0.059	0.03	0.06	0.07	−	0.11 ± 0.01
Antimony (Sb)	0.00	-	-	0.01	0.04	0.03	−	−
Cobalt (Co)	< 1	0.1843	-	< 1	<1	<1	−	0.03 ± 0.00
Thallium (Tl)	<0.01	0.0028	-	< 0.01	<0.01	< 0.01	−	−

“–” indicates that the value is not available.

**TABLE 4:** Amino acid composition in various parts of *S. aromaticum*.

Amino acid composition	Buds		Fruits	Branches	Leaves
	(mg/kg) (11)	(mg/kg) (12)	(mg/kg) (11)	(mg/kg) (11)	(mg/kg) (11)
Aspartic acid	111.6	42.8	105.4	–	–
Serine	69.8	80.5	41.5	57.9	37.9
Glutamic acid	93.8	91.3	74.1	64.2	66.4
Glycine	61.2	–	42.3	40.5	41.4
Histidine	121.6	–	118.8	121.2	120.6
Arginine	133.1	113.7	96.1	250.1	89.9
Threonine*	38.4	264.0	40.1	–	34.8
Alanine	94.5	–	93.8	52.3	55.2
Tyrosine	77.5	40.0	69.3	64.1	66.7
Valine*	65.9	106.1	50.2	45.7	44.9
Methionine*	63.3	14.1	62.8	–	–
Lysine*	68.9	–	68.5	68.2	66.8
Isoleucine*	59.8	16.8	53.1	–	–
Leucine*	61.8	27.7	56.8	–	–
Phenylalanine *	75.0	21.1	74.8	–	83.3
Proline	154.9	–	203.7	97.6	63.7
Tryptophan*	–	12.1	–	–	–
Total amino acid (TAA)	1 351.1	830.2	1 251.2	861.8	771.6
Essential amino acid (EAA) *	433.1	461.9	406.2	113.9	229.8

“–” indicates that the value is not available. *Indicates Essential amino acid.

**TABLE 5:** Vitamin composition of *S. aromaticum* buds.

Vitamins	Buds	
	(mg/100g) (12)	(mg/100g) (13)
A	177.21 ± 3.25	–
B_1_	0.04 ± 0.01	–
B_2_	1.75 ± 0.04	–
B_3_	173.26 ± 2.33	–
B_6_	134.18 ± 2.49	0.59
C	7.31 ± 0.34	80.8
E	7.11 ± 0.52	8.52
K	−	141.8

“–” indicates that the value is not available.

### Conventional nutrients composition

The fruits contain the highest amount of total carbohydrate content. The buds contain the highest amount of crude fat. The leaves contain the highest protein content. The branches contain the highest amount of fiber. Table 1 lists the conventional nutrients present in various parts of *S. aromaticum*.

### Fatty acid composition

The fatty acid composition is similar in flower buds, fruits and branches. The leaves contain many fatty acids, and the number of fatty acids is 14. The contents palmitic, stearic, linoleic and linolenic acid were high in all parts. The higher proportion of polyunsaturated fatty acids in buds and seeds, is made up of α-linolenic acid and linoleic acid. α-Linolenic acid is one of the essential nutrients, with anti-inflammatory, anti-thrombotic, anti-arrhythmic, anti-cancer, lower blood pressure and other effects (17). Linoleic acid can be metabolized into arachidonic acid in the body, to play a pro-inflammatory or pro-thrombotic vasoconstriction (18). The highest proportion of saturated fatty acids present in branches amounts to 73.66%. The palmitic acid content in saturated fatty acids is about 47.69%. The palmitic acid can play an antitumor role by activating Saos-2 cell apoptosis through endoplasmic reticulum stress and autophagy (19). Thus *S. aromaticum* is rich in fatty acids and has broad prospects for preparing health supplements. The fatty acid composition in each part of *S. aromaticum* is shown in Table 2.

### Mineral elements composition

About 26 mineral elements have been detected in *S. aromaticum*. Fe is highest in the fruits, and Ca in the leaves. The total mineral element content in buds is higher than in other parts. The mineral element content of each part of *S. aromaticum* is shown in Table 3.

### Amino acid composition

Amino acids are a combination of two organic substances, basic amino and acidic carboxyl groups, which give biochemical activity to protein molecules and are important components of proteins. The buds and fruits have similar percentages of essential amino acids, with total contents of 433.1–461.9 mg/kg and 406.2 mg/kg (32. 46%), respectively, which are higher than branches (113.9 mg/kg) and leaves (229.8 mg/kg). The details are shown in Table 4.

### Vitamins composition

Vitamins are essential nutrients for human health. The buds contain a variety of vitamins (Table 5), among which Vitamin A, B_3_, and Vitamin B_6_ are high. These vitamins promote bone development, protect eye vision, maintain normal skin function, maintain immunity, and promote blood red blood cell metabolism (20).

## Phytochemical composition

At present, it is believed that the chemical compositions of *S. aromaticum* are divided into two major parts: volatile and non-volatile components, mainly including aromatics, sesquiterpenoids, monoterpenoids, flavonoids, triterpenoids, organic acids, etc. These chemical components are mainly derived from flower buds, leaves, seeds, and other parts of *S. aromaticum*.

### Volatile components

The volatile components of *S. aromaticum* have a unique and attractive fragrance. They can be used as a topical application to relieve pain and promote healing. They are also used in perfumey and flavor industries. They possess significant antioxidant and antibacterial effects. Studies have confirmed the presence of volatile components in buds, seeds and leaves, with the majority in buds. More than 110 compounds are present in the *S. aromaticum* volatile oil and are listed in Supplementary Table 1. The major compounds in volatile oil are eugenol, eugenyl acetate, caryophyllene and α-humulene.

#### Aromatics

Aromatic compounds (eugenol, isoeugenol and eugenyl acetate) are the main components in *S. aromaticum*. Eugenol, is a unique and widely studied volatile component of *S. aromaticum*, accounting for more than 50% of the volatile oil, with a variety of pharmacological activities (31). Eugenyl acetate, is another bioactive component of the volatile components. Forty-two aromatic compounds, including methyl eugenol, ethyl benzoate, and cinnamic aldehyde, were identified in the essential oil of clove by GC-MS.

#### Terpenoids

The terpenoids in *S. aromaticum* include sesquiterpenes, monoterpenes, and diterpenes, which are the material basis for the clinical efficacy and an important chemical source for further activity screening. Terpenoids are an important class of natural compounds widely used in the cosmetics and food industries and have a large market potential (32). Twenty-six sesquiterpenes and eighteen monoterpenes were reported in the volatile oil of *S. aromaticum*. Only one diterpene, menthyl chavicol, was reported from *S. aromaticum*. Caryophyllene and α-humulene account for about 7-16% of the volatile oil.

#### Aliphatic compounds

*S. aromaticum* volatile oil also contains aliphatic compounds, such as alkanes, ketones, alcohols, esters and ethers. The most abundant aliphatic compounds are tritetracontane, 2-heptanone, 2-non-anone, ethyl caproate and menthyl octanoate. The percentage of aliphatic compounds is lower than aromatic compounds in the volatile oil.

### Non-volatile components

A total of 73 non-volatile components were reported from *S. aromaticum*, including 36 flavonoids, 4 chromones, 26 tannins, 3 triterpenoids, 1 coumarin and 1 phenolic ester, and 2 other components, as shown in Table 6.

**TABLE 6:** Composition of non-volatile components of different parts of *S. aromaticum*.

Classes	Chemical compound	Molecular formula	Extraction solvent	Biological activity	References
**Flower buds**					
Flavonoid glycosides	Rhamnetin-3-O-β-D-glucuronide	C_22_H_20_O_13_	70% Ethanol, Ethyl acetate	Antitumor	(33)
	Rhamnazin-3-O-β-D-glucuronide	C_23_H_22_O_13_	70% Ethanol, Ethyl acetate	Antitumor	(33)
	Rhamnazin-3-O-β-D-glucuronide-6′′-methyl ester	C_24_H_24_O_13_	70% Ethanol, Ethyl acetate	Antitumor	(33)
	Rhamnocitrin-3-O-β-D-glucuronide-6′′-methyl ester	C_23_H_22_O_12_	70% Ethanol, Ethyl acetate	Antitumor	(33)
	Quercetin-3-O-β-D-glucuronide	C_21_H_18_O_13_	70% Ethanol, Ethyl acetate	Antitumor	(33)
	Isorham-netin-3-O-β-D-glucuronide	C_22_H_20_O_13_	70% Ethanol, Ethyl acetate	Antitumor	(33)
	Kaempferol-3-O-β-D-glucuronide-6′′-methyl ester	C_22_H_20_O_12_	70% Ethanol, Ethyl acetate	Antitumor	(33)
	Quercetin-3-O-β-D-glucuronide-6′′- methyl ester	C_22_H_20_O_13_	70% Ethanol, Ethyl acetate	Antitumor	(33)
	Isorhamnetin-3-O-β-D-glucuronide-6′′-methyl ester	C_23_H_22_O_13_	70% Ethanol, Ethyl acetate	Antitumor	(33)
	Kaempferol-3-O-β-D-glucoside	C_21_H_20_O_11_	70% Ethanol, Ethyl acetate	Antitumor	(33)
	Quercetin3-O-β-D-glucoside	C_21_H_20_O_12_	70% Ethanol, Ethyl acetate	Antitumor	(33)
	Isorhamnetin-3-O-β-D-glucoside	C_21_H_22_O_12_	70% Ethanol, Ethyl acetate	Antitumor	(33)
	Rhamnazin-3-O-β-D-glucoside	C_23_H_24_O_11_	70% Ethanol, Ethyl acetate	Antitumor	(33)
	Quercetin-7-O-β-D-glucoside	C_21_H_20_O_12_	70% Ethanol, Ethyl acetate	Antitumor	(33)
	5,7-Dihydroxy-2-methylchromone 8-C-β-D-glucopyranoside	–	Methanol	Antibacterial	(34)
	4′-*O*-Methyl—epigallocatechin 7-*O*-glucuronide	C_22_H_24_O_13_	70% Ethanol in Milli-Q water with 0.1% formic acid	Antioxidant	(35)
	3′-*O*-Methyl-(-)-epicatechin 7-*O*-glucuronide	C_22_H_24_ O_12_	70% Ethanol in Milli-Q water with 0.1% formic acid	Antioxidant	(35)
	Hesperetin 3′-*O*-glucuronide	C_22_H_22_O_12_	70% Ethanol in Milli-Q water with 0.1% formic acid	Antioxidant	(35)
Flavonoids	Luteolin	C_15_H_10_O_6_	70% Ethanol, Ethyl acetate	Antitumor, Neuroprotective	(33)
	Quercetin	C_15_H_10_O_7_	70% Ethanol, Ethyl acetate	Antitumor, Antioxidant	(33)
	Rhamnocitrin	C_16_H_12_O_6_	70% Ethanol, Ethyl acetate, Methanol	Antitumor, Antibacterial	(33, 34)
	Kumatakenin	C_17_H_14_O_6_	70% Ethanol, Ethyl acetate	Antitumor	(33)
	Pachypodol	C_18_H_16_O_7_	70% Ethanol, Ethyl acetate	Antitumor, Antibacterial	(33)
	Kaempferol	C_15_H_10_O_6_	Methanol	Antibacterial, Antitumor, Anti-inflammatory	(34)
	Myricetin	C_15_H_10_O_8_	Methanol	Antibacterial, Antitumor, Antiviral	(34)
	Isorhamnetin	C_16_H_12_O_7_	70% Ethanol in Milli-Q water with 0.1% formic acid	Antioxidant, Antitumor, Anti-inflammatory	(35)
	3′-Hydroxygenistein	C_15_H_10_O_6_	70% Ethanol in Milli-Q water with 0.1% formic acid	Antioxidant	(35)
	4′-Methoxy-2′,3,7-trihydroxyisoflavanone	C_16_H_14_O_6_	70% Ethanol in Milli-Q water with 0.1% formic acid	Antioxidant	(35)
	3′,4′,7-Trihydroxyisoflavanone	C_15_H_12_O_5_	70% Ethanol in Milli-Q water with 0.1% formic acid	Antioxidant	(35)
	3′-Hydroxymelanettin	C_16_H_12_O_6_	70% Ethanol in Milli-Q water with 0.1% formic acid	Antioxidant	(35)
	Violanone	C_17_H_16_O_6_	70% Ethanol in Milli-Q water with 0.1% formic acid	Antioxidant	(35)
	2-Dehydro-*O*-desmethylangolensin	C_15_H_12_O_4_	70% Ethanol in Milli-Q water with 0.1% formic acid	Antioxidant	(35)
	5-Hydroxy-4′-methoxy-6,7-methylenedioxy Isoflavone	C_17_H_12_O_6_	Petroleum ether–Ethyl acetate	–	(36)
	5,4′-Dimethoxy-6,7-methylenedioxy Isoflavone	C_18_H_14_O_6_	petroleum ether-Ethyl acetate	–	(36)
Chromones	Eugenin	C_11_H_10_O_4_	Ethyl acetate soluble fraction of the Methanol	Anticholinesterase	(37)
Chromone glycosides	8-C-glucosylnoreugenin	C_16_H_18_O_9_	Ethyl acetate soluble fraction of the Methanol	Anticholinesterase	(37)
	Biflorin	C_16_H_18_O_9_	Methanol, Ethyl acetate	Anti-inflammatory, Antiviral, Antitumor	(38, 39)
	Isobiflorin	C_16_H_18_O_9_	Methanol, Ethyl acetate	Anti-inflammatory, Antiviral, Antitumor	(38, 39)
Tannins	Gallic acid	C_7_H_6_O_5_	Methanol, Ethyl acetate soluble fraction of the Methanol	Antibacterial, Anticholinesterase	(34, 37, 40)
	Ellagic acid	C_14_H_6_O_8_	Methanol	Antibacterial, Antioxidant	(34)
	Gallic acid 4-*O*-glucoside	C_13_H_16_O_10_	70% Ethanol in Milli-Q water with 0.1% formic acid	Antioxidant	(35)
	Paeoniflorin	C_23_H_28_O_11_	70% Ethanol in Milli-Q water with 0.1% formic acid	Antioxidant, Anti-inflammatory, Immunoregulatory, Neuroprotective	(35)
	3-*p*-Coumaroylquinic acid	C_16_H_18_O_8_	70% Ethanol in Milli-Q water with 0.1% formic acid	Antioxidant	(35)
	Eugeniin	C_41_H_30_O_26_	Methanol, Ethyl acetate	Antiviral	(39)
	Casuarictin	C_41_H_28_O_26_	Methanol, Ethyl acetate, 70% Acetone	Antiviral, Antifungal	(39, 40)
	1,3-Di-O-galloyl-4,6-(S)-hexahydroxy-diphenoyl-β-D-glucopyranose	C_33_H_23_O_22_	Methanol, Ethyl acetate	Antiviral	(39)
	Tellimagrandin I	C_33_H_23_O_22_	Methanol, Ethyl acetate	antiviral	(39)
	1,2-Di-O-galloyl-3-O-digalloyl-4,6-O-(S)-hexahydroxydiphenoy-β-D-glucose	C_34_H_20_O_22_	Methanol, Ethanol, 70% Acetone	Antifungal	(40)
	Tellimagrandin II	C_41_H_30_O_26_	Methanol, Ethanol, 70% Acetone	Antifungal	(40)
	Aromatinin A	C_48_H_32_O_30_	Methanol, Ethanol, 70% Acetone	Antifungal	(40)
	Syzyginin A	C_48_H_34_O_31_	Methanol, Ethanol, 70% Acetone	Antifungal	(40)
	Bicornin	C_48_H_32_O_30_	Methanol, Ethanol, 70% Acetone	Antifungal	(40)
	Platycaryanin A	C_48_H_32_O_31_	Methanol, Ethanol, 70% Acetone	Antifungal, Anti-inflammatory	(40)
	Alunusnin A	C_47_H_25_O_30_	Methanol, Ethanol, 70% Acetone	Antifungal	(40)
	Rugosin C	C_47_H_32_O_30_	Methanol, Ethanol, 70% Acetone	Antifungal	(40)
	1,2,3-Tri-O-galloyl-β-D-glucose	C_24_H_37_O_24_	Methanol, Ethanol, 70% Acetone	Antifungal	(40)
	1,2,3,6-Tetra-O-galloyl-β-D-glucose	C_30_H_47_O_12_	Methanol, Ethanol, 70% Acetone	Antifungal	(40)
	Heterophylliin D	C_82_H_54_O_52_	Methanol, Ethanol, 70% Acetone	Antifungal	(40)
	Rugosin D	C_82_H_58_O_52_	Methanol, Ethanol, 70% Acetone	Antifungal	(40)
	Rugosin F	C_82_H_56_O_5_	Methanol, Ethanol, 70% Acetone	Antifungal	(40)
	Euprostin A	C_54_H_29_O_34_	Methanol, Ethanol, 70% Acetone	Antifungal	(40)
	Alienanin B	C_72_H_52_O_51_	Methanol, Ethanol, 70% Acetone	Antifungal	(40)
	Squarrosanin A	C_55_H_32_O_33_	Methanol, Ethanol, 70% Acetone	Antifungal	(40)
	Casuarinin	C_41_H_28_O_26_	Methanol, Ethanol, 70% Acetone	Antioxidant, Antifungal, Antitumor	(40)
Triterpenoids	Oleanolic acid	C_30_H_48_O_3_	Methanol	Antibacterial	(34)
	Asiatic Acid	C_30_H_48_O_5_	Dichloromethane–Methanol	Antioxidant, Antitumor	(36)
	Arjunolic Acid	C_30_H_48_O_5_	Dichloromethane–Methanol	Antioxidant, Antifungal, Antibacterial, Anticholinesterase, Antitumor	(36)
Coumarins	Scopoletin	C_10_H_8_O_4_	70% Ethanol in Milli-Q water with 0.1% formic acid	Antioxidant, Antitumor	(35)
Phenolic acid esters	Salvianolic acid C	C_26_H_20_O_10_	70% Ethanol in Milli-Q water with 0.1% formic acid	Antioxidant, Anti-inflammatory, Antitumor	(35)
Others	2,6-Dihydroxy-4-methoxyacetophenone 3-C-β-D-glucoside	C_15_H_19_O_9_	Ethyl acetate soluble fraction of the Methanol	Anticholinesterase	(37)
	2,6-Dihydroxy-4-methoxyacetophenone 3-C-β-D-(6′-O-galloyl) glucoside	C_22_H_23_O_13_	Ethyl acetate soluble fraction of the Methanol	Anticholinesterase	(37)
Seeds					
Flavonoid glycosides	Apigenin 6-C-[β-D-xylopyranosyl-(1→2′′)-β-D-galactopyranoside]-7-O-β-D-glucopyranoside	C_32_H_38_O_19_	Ethanol	–	(41)
	Apigenin 6-C-[β-D-xylopyranosyl-(1→2′′)-β-D-galactopyranoside]-7-O-β-D-(6′′′-O-p-coumarylglucopyranoside)	C_41_H_44_O_21_	Ethanol	–	(41)

“–” indicates that the item is not retrieved.

#### Flavonoids

Studies have shown that flavonoids are one of the important components of *S. aromaticum* to exert strong antioxidative activity, anti-inflammatory, and immunomodulatory activities (42). Ryu et al. (33) reported the presence of luteolin, quercetin, rhamnocitrin, kumatakenin, and pachypodol in *S. aromaticum*. These flavonoids have shown anticancer activity on human ovarian cancer cells (A2780). In addition, flavonoids readily form O - and C - glycosides, with O - glycosides being more common and better absorbed. Ryu et al. (33) reported nine known flavonoid glycosides and four flavonoids from the buds.

#### Chromones

The chromones in *S. aromaticum* are mainly eugenin, 8-C-glucosylnoreugenin, biflorin, isobiflorin, etc. Biflorin and isobiflorin were found to be isolated from *S. aromaticum* buds by Lee et al. (38) and showed an anti-inflammatory effect on Lipopolysaccharide (LPS) induced inflammation in macrophages through STAT1 inactivation. In addition, it was demonstrated that biflorin increased the activation of protein kinase C-ζ and its downstream signaling molecules in the hippocampus. These compounds improved the cognitive dysfunction in mice and reduced the viability of melanoma cell lines through DNA interactions (43). These findings provide a scientific basis for the neuroprotective and antitumor effects of *S. aromaticum*.

#### Tannins

Tannins are a group of water-soluble polyphenolic compounds with complex structures present in plants. Based on the chemical structures, these can be classified into two major groups, hydrolyzable and condensed tannins. The tannins exhibit various pharmacological effects such as antibacterial, antioxidant, antitumor, antiviral, and hypoglycemic (44). Kim et al. (39) isolated four tannins from *S. aromaticum*, eugeniin, casuarictin, 1,3-di-O-galloyl-4,6-(S)-hexahydroxydiphenoyl-β-D-glucopyranose, and tellimagrandin I. The tellimagrandin was found to have significant inhibitory activity on syncytium formation. Ali et al. (35) analyzed the polyphenol and tannin content in 12 spices (allspice, black cardamom, black cumin, black pepper, cardamom, cinnamon, clove, cumin, fennel, nutmeg, star-anise, and turmeric) using LC-ESI-QTOF-MS and evaluated their antioxidant activity. Cloves contain the highest total polyphenol and total tannin content. The antioxidant activity is positively correlated with the total phenolic content. *S. aromaticum* is the most active antioxidant (45).

#### Others

Triterpenoids (asiatic acid, arjunolic acid and oleanolic acid), coumarins (scopoletin), and phenolic acid esters (salvianolic acid C) were reported from *S. aromaticum*. Oleanolic acid, has shown significant analgesic effects. The oleanolic acid activates the bile acid receptor (TGR5), which plays a key role in treating metabolic diseases. The acetyl derivatives of oleanolic acid have shown better anti-inflammatory activity than oleanolic acid (46).

## Biological and pharmacological activities

Different parts of *S. aromaticum* and its essential oil have various pharmacological activities, including antioxidant, hypoglycemic, antitumor, antibacterial antiviral, etc. The majority of the pharmacological investigations were mainly focused on the flower buds (clove). The details were discussed in each of the following paragraphs, and are recapitulative summary was presented in Table 7.

**TABLE 7:** The biological effects of *S. aromaticum*.

Biological effects	Part	Extract/ Compound	Assay	Model and effective concentrations	Reference
Antioxidant activity	Buds, Flower, Seeds, Leaves	Eugenol	ABTS, DPPH, SDS-PAGE and Western blotting analysis	Neutrophils (IC_50_: 5 μg/mL)	(47)
	Buds	Aqueous extract	DPPH, TEAC, FRAP, ORAC, HRSA, SRSA	IC_50_: 0.29 ± 0.01 mg/ml	(48)
	Leaves	70% Ethanol extract	Spot assay, Mitochondrial, ROS Levels	Yeast Schizosaccharomyces pombe cell (100 ppm)	(49)
Hypoglycemic activity	Buds	50% Aqueous EtOH extract	Blood glucose, HbA1c levels	db/db Mouse (IC_50_: 4.7 μM)	(50)
	Buds	-	Blood glucose, Serum insulin, Insulin receptor and Leptin levels	Male rabbits [12.5% (w/w)]	(51)
Antitumor activity	Buds	Ethyl acetate extract	Western blot and (qRT)-PCR analysis	HT-29 tumor xenograft model (IC_50_: 66 ± 8 μg/ml)	(52)
	Buds	Methanolic extract	Sulforhodamine-B assay	Uterine cervix (GI _50_: 36 μg/ml), Breast (GI _50_: 50 μg/ml), Lung NCI (GI _50_: 68 μg/ml)	(53)
Antibacterial activity	Leaves	Steam distillation method	Time-kill curve assay, Scanning electron microscopy assay	Porphyromonas gingiva (MIC: 6.25 μM)	(54)
	Seeds	Aqueous extract	Dilution method (agar), Time-kill assay	Escherichia coli, Pseudomonas aeruginosa and Staphylococcus aureus (MIC: 0.06 mg/mL, MBC:0.10 mg/mL)	(55)
	Buds	Steam distillation method	Disk diffusion method, Broth microdilution method, Scanning electron microscopy	Escherichia coli and Klebsiella pneumoniae [MIC: 0.078% (v/v)]	(56)
Immunomodulatory, anti-inflammatory, and analgesic activities	Buds	Steam distillation method	Enzyme-linked immunosorbent assay, Sulforhodamine B	Human dermal fibroblast system HDF3CGF [0.011% (v/v)]	(57)
	Buds	Alcoholic extraction and distilled water	MTT, Enzyme-linked immunosorbent assay	Female inbred Balb/c mice (100 μg mL and 1000 μg/mL)	(58)
Neuroprotective activity	Buds, Flower, Seeds, Leaves	Eugenol	Y maze alternation, Novel Object Recognition, Morris Water Maze, FD Rapid Golgi StainingTM, Hematoxylin-Eosin staining	Male Deutchland Denken Yonken mice (100 mg/kg bw)	(59)
Antiviral activity	Buds, Flower, Seeds, Leaves	Eugenol	Medium and high-throughput screens, Hoechst stains, MTT	Ebola virus (EC_50_: 1.3 μM)	(60)
	Buds	95% Ethanol	Plaque reduction assay	Herpes simplex virus type 1 (ED_50_: 72.8 μg/mL), Herpes simplex virus type 2 (ED_50_: 74.4 μg/mL)	(61)
Antiobesity activity	Buds	70% Ethanol	Body weight, Hematoxylin and eosin stain, Western blotting	Male C57BL/6J mice [0.5% (w/w)]	(62)

### Antioxidant activity

Clove essential oil (CEO) has shown powerful antioxidant activity with an EC_50_ of 0.36 μL/mL and it is the most potent volatile oil compared to eucalyptus, fennel and lavender (63). Phenols and flavonoids were attributed to antioxidant effects. The antioxidant activity is mediated via scavenging the free radicals, ferric reducing capacity, increasing the activity of antioxidant enzymes, and antagonizing lipid peroxidation (LPO).

Baghshahi et al. (64) showed that the antioxidant activity of clove extract (CE) was more than 10 times higher than that of vitamin E in the DPPH free radical scavenging capacity test. Reactive oxygen species (ROS) are oxygen-containing intermediate metabolites that modulate the host’s immune response *in vivo* and positively affect the clearance of dead cells and the inactivation of microorganisms, but excessive amounts of ROS *in vivo* can damage the organism at the cellular level. Neutrophils are the most abundant leukocytes in humans, and their hyperactivation can activate the NADPH oxidase to generate large amounts of ROS. Eugenol inhibits fMLF or PMA-induced superoxide anion production in neutrophils by inhibiting the Raf/MEK/ERK1/2/p47phox phosphorylation pathway, avoiding ROS accumulation (47). CEO can increase the activity of antioxidant enzymes by inducing the expression of SOD-3 or GST-4 to reduce ROS accumulation *in vivo*, exerting antioxidant effects (65). Eugenol also has Fe^3+^ reducing ability and electron donor properties, which can neutralize free radicals by forming stable products to exert antioxidant effects (66).

One of the common oxidative reactions, LPO, is associated with many diseases. DNA damage caused by ROS is a key factor in tumorigenesis and development. Eugenol can effectively block hydroxyl radical-induced DNA oxidation and LPO and has a significantly higher inhibitory effect on hydrogen peroxide than other reactive oxygen species (67). CEO also inhibits LPO in erythrocyte membranes, thereby increasing membrane resistance to spontaneous hemolysis, reducing membrane microviscosity, maintaining its structural integrity and functional activity, and significantly decreasing the intensity of LPO in mouse liver and brain, scavenging excess ROS and other free radicals from lipid chains, increasing the antioxidant capacity of liver and brain lipids and the activity of antioxidant enzymes in the liver (68). CE also significantly prevented oxidation-induced protein damage by reducing the formation of protein carbonyl groups and preventing the loss of protein sulfhydryl groups (48).

**FIGURE 3:**
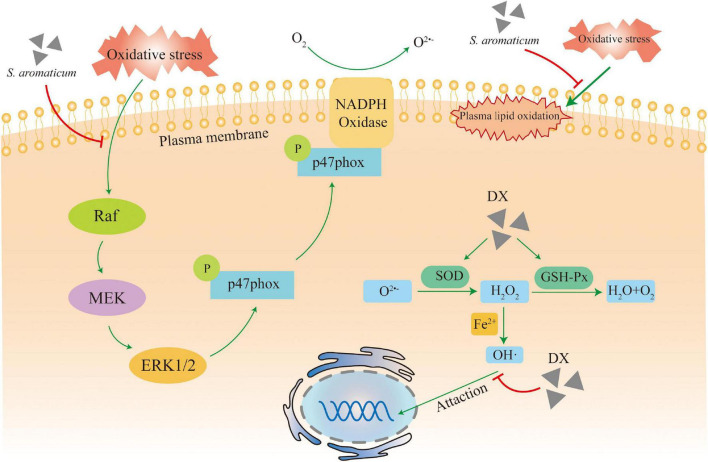
Mechanism of action for the antioxidant activity.

In conclusion, *S. aromaticum* can reduce free radical accumulation *in vivo*, decrease oxidative cellular damage, and increase antioxidant capacity (Figure 3). Thus, it has very high potential to be used as a natural antioxidant and anti-aging supplement.

### Hypoglycemic activity

Abnormal glucose metabolism leads to complications of various metabolic diseases, especially the prevalence of diabetes is increasing worldwide, but commonly used oral hypoglycemic drugs often cause serious side effects. So herbs and spices have been used in folk medicine for centuries to control the complications of diabetes. *S. aromaticum* has been widely studied for its beneficial and toxic effects. CE has shown comparable hypoglycemic effects to that of insulin in animal models and did not show toxic effects (69).

Abdulrazak et al. (51) found a significant increase in insulin and leptin levels in type 2 diabetic rabbits treated with 12.5% clove for 6 weeks suggesting its potential to use for patients with diabetes. Some studies have shown that CE and its compound nigricin enhance proximal insulin signaling by decreasing serine phosphorylation of insulin receptor substrate-1 (IRS-1), increasing tyrosine phosphorylation, modifying distal insulin signaling by enhancing protein kinase B (PKB) and glycogen synthase kinase-3-β (GSK-3β) phosphorylation in muscle cells signaling. Thus, they decrease free fatty acid-mediated insulin resistance in mouse myogenic cells, increase glucose uptake, and promote insulin secretion in muscle cells (70).

Glycogen metabolism has been one of the main causes of elevated blood glucose levels. Glycogen phosphorylase b (PYGB), a key enzyme in glycogen degradation, catalyzes the breakdown of glycogen to glucose-1-phosphate in the liver and skeletal muscle. Therefore, inhibition of hepatic glycogen phosphorylase is an effective therapeutic strategy to reduce hyperglycemia in type 2 diabetes. The clove’s 50% aqueous ethanol extract showed strong dose-dependent inhibitory activity on glycogen phosphorylase b and glucagon-stimulated gluconeogenesis in primary rat hepatocytes, significantly suppressed blood glucose and glycated hemoglobin (HbA1c) levels in db/db mice. Thus, it caused a significant reduction in plasma triglyceride and non-esterified fatty acid levels, and improved glucose and lipid metabolism (50). In addition, the dehydrodienol and dehydrodienol B compounds of clove ethanol extracts (ECE) have potent human peroxisome proliferator-activated receptor (PPAR)-γ ligand binding activity, which can stimulate 3T3-L1 preadipocyte differentiation through PPAR-γ activation and significantly inhibit the increase in blood glucose levels in type 2 diabetic KK-Ay mice (71). Studies on the effects of C2C12 cardiomyocyte metabolism revealed that CE increased the phosphorylation of AMP-activated protein kinase (AMPK) and its substrate acetyl coenzyme A carboxylase (ACC), and also transcriptionally regulated genes involved in metabolism, including sirtuin1 (SIRT1) and PPARγ coactivator 1α (PGC1α), as a way to increase muscle glycolysis and mitochondrial function (69).

**FIGURE 4:**
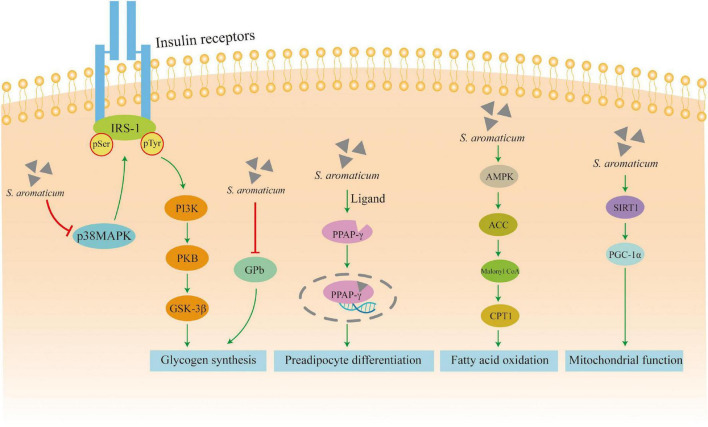
Mechanism of action for the hypoglycemic activity.

Overall, *S. aromaticum* has the potential to exert type 2 diabetes through phosphorylation pathways that modify insulin signaling, activation of receptor PPAR-γ ligand binding activity, and activation of AMPK and SIRT1 pathways (Figure 4).

### Antitumor activity

Tumor pathogenesis is complex, and impaired apoptosis mechanism is a major cause. So selective induction of apoptosis in tumor cells is one of the effective ways to treat tumors. *S. aromaticum* has anticancer and antitumor activity on the human colon, breast, liver, cervical, and gastric cancers. Its inhibitory effects are time- and dose-dependent. Arung et al. (72) found that CEO inhibited melanin in B16 melanoma cells up to 50% and 80% at 100 and 200 μg/mL, respectively. Ethyl acetate extract of clove and its active ingredient oleanolic acid both selectively increased the protein expression of p21(WAF1/Cip1) and γ-H2AX and downregulated the expression of cell cycle regulatory proteins, promoted G_0_/G_1_ cell cycle arrest and induced apoptosis in a dose-dependent manner. Its activity on colon tumor xenografts is superior to the chemotherapeutic drug 5-fluorouracil (52). PI3K/Akt/mTOR signaling pathway is an important pathway that regulates apoptosis and can be aberrantly activated by malignant tumor cells to exert apoptosis-inhibiting and proliferation-promoting effects in tumor cells. The active ingredients of clove inhibit PI3K/Akt/mTOR signaling pathway and activate the caspase-mediated cascade response to induce apoptosis of HCT116 cells in a dose-dependent manner (73).

Bcl-2 family proteins play a key role in apoptosis and play an important role in mitochondrial-mediated apoptosis by regulating mitochondrial outer membrane permeability. The study has shown that CE can induce endogenous caspase-dependent cell death by increasing oxidative stress mediated via oxygen and nitrogen radicals. It can mediate the release of Bcl-2 family protein pro-apoptotic factors, signaling oxidative stress-mediated DNA damage by modulating the cellular antioxidant SOD system and the activity of the Akt, p38mitogen-activated protein kinases (p38MAPK), c-Jun N-terminal kinase (JNK) and extracellular signal-regulated kinases (Erk1/2) pathways to induce apoptosis in human breast cancer MCF-7 cells (74). The aqueous extract of clove (ACE) upregulated the expression of pro-apoptotic proteins p53 and Bax in benzo[a]pyrene (BP)-induced lung carcinogenesis in mice. It downregulated the expression of anti-apoptotic protein Bcl-2 in the pre-cancerous stage. In the early stage of carcinogenesis (week 8), clove significantly activated the expression of caspase-3. In addition, clove downregulated the expression of COX-2, cMyc, HRA and other growth-promoting proteins expression. All these together promote a significant decrease in proliferating cells and an increase in apoptotic cells, exerting a chemopreventive potential (75). In addition, eugenol can also exert its chemotherapeutic potential by blocking the nuclear translocation of β-catenin, thus promoting its cytoplasmic degradation through N-terminal phosphorylation of Ser37. Thus, it effectively reduces cancer complications and prolongs and improves patient life (76).

**FIGURE 5:**
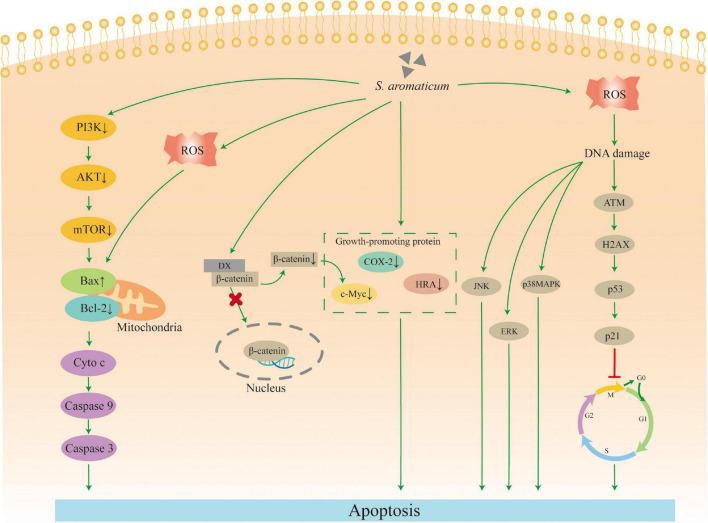
Mechanism of action for the antitumor activity.

In conclusion, the main pathway of *S. aromaticum* to induce apoptosis in cancer cells by upregulating the expression of intracellular pro-apoptotic proteins such as Bax, Caspase-3, p53, etc., downregulating the expression of intracellular anti-apoptotic proteins such as Bcl-2, and downregulating the expression of pro-growth proteins such as COX-2, cMyc, and HRA (Figure 5).

### Antibacterial activity

In recent years, the irrational application of broad-spectrum antibacterial drugs, immunosuppressive drugs, antitumor drugs and the widespread use of other surgical interventions such as organ transplantation have led to an increasingly serious crisis of bacterial and fungal drug resistance, which threatens the life and health of humans. Clove has strong antibacterial effects on *Staphylococcus aureus*, *Candida albicans*, *Escherichia coli* and *Listeria monocytogenes*.

It was found that ACE and ECE showed inhibitory activity against three foodborne pathogens, gram + ve *Staphylococcus aureus*, gram -ve *Escherichia coli* and *Pseudomonas aeruginosa*. *S. aureus* is the most sensitive to ACE (inhibition zone, 30.5 mm), *P. aeruginosa* is the most sensitive to ECE (inhibition zone, 38 mm). ACE inhibited the growth of *S. aureus* and *E. coli* at ≥500 μg/ml, *P. aeruginosa* at ≥700 μg/ml, respectively. ECE inhibited the growth of all three bacteria at ≥500 μg/ml (77). Similarly, Ajiboye et al. (55) found that aqueous extracts of clove seeds could enhance the membrane permeability of *Escherichia coli*, *Pseudomonas aeruginosa*, and *Staphylococcus aureus*, exerting antibacterial effects. In addition, four different CE (methanol, ethyl acetate, n-hexane and ether) showed inhibitory activity against *Candida albicans, Candida glabrata* and *Candida tropicalis*, with the ethyl acetate extract being the most active. CEO also antagonized *Candida biofilm* formation and effectively prevented the growth of *Candida* on abiotic surfaces (24). In addition, Zhang et al. (54) found that the eugenol in *S. aromaticum* leaf essential oil exhibited good antibacterial activity against *Porphyromonas gingivalis* at a concentration of 31.25 μm.

Xu et al. (78) showed that the antibacterial activity of *S. aromaticum* is via disrupting the cell wall and membrane, inhibiting the normal synthesis of bacterial DNA and proteins, eugenol is the main component in the antibacterial activity. Quorum sensing (QS) is a communication system associated with the virulence of pathogenic bacteria such as *Pseudomonas aeruginosa*. The molecular modeling studies have shown that eugenol binds to the QS receptor overcoming the antibiotic resistance through hydrophobic interactions and hydrogen bonding to Arg61 and Tyr41 of the LasR receptor (79). Elastase A, elastase B, proteinase IV and alkaline proteases activate host matrix metalloproteinases (MMPs) to establish infection by converting pre-MMP-2 to active MMP-2. *S. aromaticum* leads to a significant reduction of signaling molecules (Acyl-homoserine lactones) involved in population-sensing regulation, which can inhibit the activity of four proteases from establishing anti-infective effects, in addition to inducing a dose-dependent production of neutrophil extracellular traps (NETs) to destroy bacterial pathogens (80). CEO interferes with the expression of virulence-related genes involved in the flagellum, PEB1, PEB4, LPS and serine protease, with significant antibacterial and potentially virulence-modulating effects on *Campylobacter jejuni* (81). eEF1A protein interacted with several viral proteins, leading to enhanced viral replication, reduced apoptosis and increased cellular transformation; therefore, Wang et al. (82) suggested that downregulation of eEF1A protein expression may be another important mechanism for exerting its antifungal activity.

**FIGURE 6:**
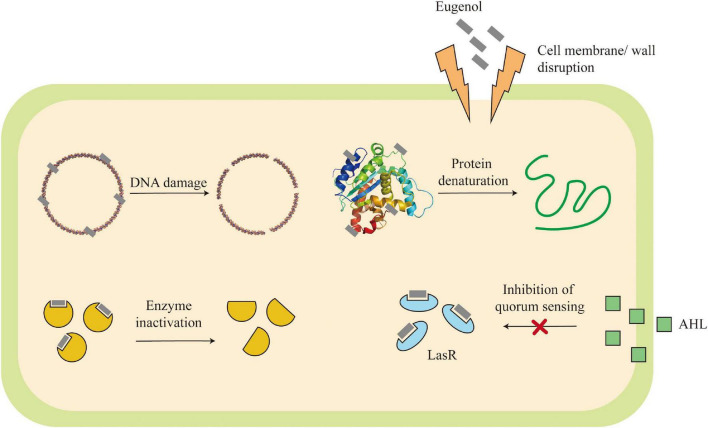
Mechanism of action for antibacterial activity.

In conclusion, the permeability of phenolic substances such as eugenol to cell membranes and the irreversible disruption of plasma membrane integrity make *S. aromaticum* a potential source of natural antibacterial drugs with broad-spectrum antibacterial effects (Figure 6).

### Immunomodulatory, anti-inflammatory, and analgesic activities

**FIGURE 7:**
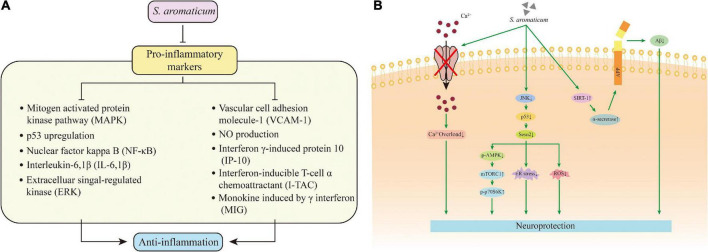
Mechanism of action for the immunomodulatory **(A)** and neuroprotective **(B)** activity.

Clove essential oil (CEO) regulates the immune response and reduces inflammatory symptoms by enhancing humoral immunity and reducing the release of lymphokines to reduce cellular immunity. As a major component of *S. aromaticum*, eugenol is thought to regulate cellular inflammatory cascades in LPS induced inflammation in macrophages *via* (1) nuclear factor-κB (NF-κB) and ERK/MAPK pathways, (2) nitric oxide (NO) production, (3) pro-inflammatory interleukin release and (4) endogenous antioxidant defenses mechanisms (83). Secondly, CEO significantly inhibited the levels of several pro-inflammatory biomarkers such as (1) vascular cell adhesion molecule-1 (VCAM-1), (2) interferon γ-induced protein 10 (IP-10), (3) interferon-inducible T-cell α chemoattractant (I-TAC), and (4) monokine induced by γ interferon (MIG)-induced monokines (Figure 7). It also significantly inhibited (1) tissue remodeling protein molecules (collagen-I, collagen-III), (2) macrophage colony-stimulating factor (M-CSF), and (3) tissue inhibitor of metalloproteinase 2 (TIMP-2). It regulated the global gene expression and altered key signaling pathways of inflammation, tissue remodeling and cancer signaling processes (57). The water-soluble fraction of the hydroalcoholic extract of clove inhibited the production of pro-inflammatory cytokines (IL-1β and IL-6) by macrophages in BALB/c mice, thus exerting an anti-inflammatory effect (84). In addition, in an immunosuppressed mouse model, CEO (400 mg/kg) administration for one week significantly increased the total white blood cell (WBC) count. It and enhanced the delayed type hypersensitivity (DTH) in mice, restoring the cellular and humoral immune responses in a dose-dependent manner in cyclophosphamide immunosuppressed mice (85).

Ferland, Beaudry, and Vachon (86) showed eugenol (40 mg/kg) reduced substance P and CGRP, and increased the dynorphin level in a rat model of osteoarthritis. These results confirmed the therapeutic potential of eugenol in osteoarthritis. CEO also reduced the torsional movements in mice with acetic acid-induced abdominal contractions and significantly increased the latency of response to pain after 60 min by 82.3%. The CEO also inhibited the foot swelling in mice caused by carrageenan by 50.6%. It has significantly attenuated yeast-induced t hyperthermia by 2.7°C at ΔT-max (87).

### Neuroprotective activity

Alzheimer’s disease (AD) is a common neurodegenerative disease characterized by progressive cognitive dysfunction and memory loss, which has been increasing in recent years, seriously affecting patients’ lives and quality of life. Oxidative stress plays a key role in AD, and CEO can reverse scopolamine-induced short- and long-term memory deficits by reducing oxidative stress (88). By administering CEO to intracerebroventricular (icv)-colchicine-induced cognitive dysfunction in rats, it was found that in addition to reducing oxidative stress damage, CEO exerted neuroprotective effects and improved cognitive dysfunction by improving mitochondrial dysfunction and inhibiting microglial activation (89). There are many links between SIRT1 and diseases such as AD. Studies have shown that ECE activates and increases SIRT1 level, inhibits NF-kB signaling in microglia, attenuates Aβ25-35-induced neuronal cell neurotoxicity, and also downregulates γ-secretase level, scavenges ROS and increases the percentage of antioxidant enzymes, which together delay the progression of neurodegenerative diseases and exert neuroprotective effects (90).

Glutamate is considered an excitatory neurotransmitter, but it causes oxidative stress at high concentrations and promotes apoptotic signaling cascades, leading to neurodegeneration. CE fermented with Trametes Versicolor (LTV), contains a higher content of dehydroeugenol. It inhibits apoptotic signaling such as Ca^2+^ inward flow, the excessive production of intracellular reactive oxygen species and LPO. It also has good protective properties against glutamate-induced toxicity in HT22 cells (91).

Rai et al. (92) reported that Sestrin2 (Sesn2) is a potential serum marker in Parkinson’s disease (PD). The Sesn2 concentrations were significantly elevated in the serum of PD patients. ECE caused a dose-dependent downregulation of p53, Sesn2 and phosphorylated AMPK in cells, accompanied by the increased phosphorylated p70S6K, alleviating SH-SY5Y cell damage and exhibiting neuroprotective effects in PD. In addition, cloves exhibit anticholinesterase activity and are protective against brain damage induced by CeCl_3_ and other substances (93).

In conclusion, *S. aromaticum* and its extracts exert neuroprotective effects by reducing acetylcholinesterase activity, restoring oxidative status, inhibiting microglia signaling and preventing histopathological changes (Figure 7).

### Antiviral activity

Studies have shown that ACE exhibits antiviral activity similar to pure eugenol on feline calicivirus (94). The plaque reduction assay demonstrated that ECE has anti-herpes simplex virus (HSV) properties, showing direct inactivation of standard HSV particles, as well as significant inhibition of HSV-1, HSV-2, and a decrease in total HSV virus yield 30 h after treatment with the extract (61). The methanol and aqueous extracts of clove also showed inhibition of HCV protease. Eugenol inhibited influenza A virus (IAV), presumably by a mechanism attributed to the inhibition of oxidative stress and activation of ERK1/2, p38MAPK, and IKK/NF-κB pathways. Eugenol inhibits Beclin1-Bcl_2_ heterodimer dissociation and autophagy, ultimately impairing IAV replication (95). In addition, eugenol showed significant inhibitory of the West African Ebola virus (EBOV) (60). It is also used for the prevention and control of SARS-CoV-2-associated diseases. Flavonoids of *S. aromaticum* can inhibit SARS-CoV-2 virus expression, while bicornin and biflorin in clove have a high affinity for M^pro^ and exhibit potential viral inhibitory activity (96).

In summary, *S. aromaticum* exerts antiviral effects, mainly by improving oxidative stress, reducing viral replication, and inhibiting autophagic gene expression.

### Anti-obesity activity

Fatty acid synthase (FAS), a key enzyme in adipogenesis, has been considered a potential therapeutic target for cancer and obesity. ECE as a FASN inhibitor can inhibit S-phase DNA replication in HepG2 cells. Adipocyte differentiation in OP9 cells, regulates the content of total triglyceride and low-density lipoprotein cholesterol, limits the development of high-fat diet-induced obesity, reduces body weight and abdominal adipose tissue weight, and reduces lipid accumulation in liver and epididymal adipose tissue, making it a potential therapeutic agent for obesity (97, 98). In addition, ECE also inhibited lipid accumulation in mice by downregulating the mRNA levels of transcription factors such as Srebp and Pparg and suppressing the expression of lipid metabolism-related proteins such as SREBP-1, FAS, CD36 and PPARγ in liver and white adipose tissue (62).

### Others activities

In addition to the above effects, *S. aromaticum* also affects reproductive function, promoting transdermal absorption, and alleviating gastric injury. CE has a bidirectional effect on reproductive function in mice, with lower doses (15 mg/kg⋅BW) of clove increasing serum testosterone levels and testicular hydroxysteroid dehydrogenase activity, and improving sperm motility, sperm morphology, epididymal and seminal vesicle secretory activity, and the number of litters per female, but higher doses (30 and 60 mg/kg⋅BW) inhibiting testicular activity (99). Choi D et al. (100) hypothesized that the mechanism affects the reproductive endocrine system by acting on GnRH neuronal cells. ACE can treat ethanol-induced gastric mucosal injury in rats. The mechanism may involve antioxidant activity, promoting PGE2 production, and inhibiting gastric mucosal inflammatory cell infiltration and epithelial cell loss (101). CEO also has significant permeation-enhancing effects, and studies on the effects of CEO on the *in vivo* and *in vitro* transdermal administration of ibuprofen in rabbits revealed that clove significantly enhanced ibuprofen absorption *in vitro* and permeation *in vivo* (102). CEO can also be used for fish anesthesia, speculating that it may act through glutamate receptors.

## Toxicity studies

The U.S. Food and Drug Administration (FDA) has confirmed the safety of clove buds, CEO, eugenol, and oleoresins as food additives. The World Health Organization (WHO) has established an acceptable daily intake of 2.5 mg/kg of CEO (6).

Some literature has reported cytotoxicity of eugenol, but it can rapidly reach peak plasma concentrations upon oral administration in rats and humans, with mean half-lives of 14.0 and 18.3 h, respectively, and is excreted in the urine in bound form within 24 h. Its genotoxicity and carcinogenicity are low (103). Acute toxicity studies in mice showed that LD_50_ of CEO is 4500 mg/kg upon oral administration for 24 h, which is a much higher dose than the doses usually used for infusions by humans (3 g/60 kg person in clinical therapy, equal to 9.35 mg/kg CEO). The long-term repeated toxicity studies (100, 200, and 400 mg/kg, orally) showed that only 400 mg/kg resulted in a significant decrease in body weight and no significant changes in relative organ weights and histopathological analysis were observed at all doses of CEO (104). Toxicity studies with clove buds polyphenol extract (Clovinol) in Wistar rats revealed no significant toxicity under acute (5 g/kg b.wt. for 14 days) and subchronic (0.25, 0.5 and 1 g/kg b.wt. for 90 days) conditions. The mutagenicity studies using *Salmonella typhimurium* strains revealed that Clovinol did not cause genetic mutations or shifts in the genome and showed significant mutagenic resistance against known mutagens, sodium azide, NPD, tobacco and 2-acetamidoflourene (105). In 2015, a panel of experts from the Association of Flavor and Extract Manufacturers initiated a re-evaluation of the safety of more than 250 natural flavor compounds (NFCs) and reported concluded that NFCs of clove was recognized as “Generally Recognized as Safe (GRAS)” under the conditions of their intended use as flavor ingredients (106). An acute oral toxicity study was conducted in normal rats according to the Organization for Economic Cooperation and Development (OECD) guidelines to assess the potential toxicity of *S. aromaticum* extracts after oral administration. Three animals per group were used in each step. Standard doses were given gradually, from 500 mg/kg b.w. continued up to 2500 mg/kg b.w. Mortality was recorded after 24 h. According to various acute and chronic toxicity studies of *S. aromaticum*, the oral LD_50_ of CEO in the food industry was 3597.5 mg/kg, with no adverse effects in subchronic toxicity tests and NAOEL levels of 900-2000 mg/kg/day. Oral LD_50_ of eugenol was reported as 2650–3000 mg/kg b.w. (105), all of which demonstrated the safety of *S. aromaticum* for human use at low doses.

## Development and applications of *Syzygium aromaticum*

### Folk medicine

As one of the traditional spices and herbs, clove has long been used to treat stomach disorders, abdominal pain, vomiting, etc. Many scientific studies have confirmed that clove possesses significant free radical scavenging and anti-inflammatory activities and has potential roles in alleviating and preventing diseases like cancer, type II diabetes and epilepsy. CEO also has a stomachic effect and can significantly improve the symptoms of loss of appetite (107). In addition, clove has long been used as a respiratory adjuvant in treating respiratory diseases such as cough, asthma, and bronchitis. It also plays an important role in preventing and early treatment of COVID-19 (108).

Because of its analgesic, anesthetic and anti-inflammatory properties, CEO is often used to treat dental diseases. The traditional treatment of toothache with CEO was first documented in 1640 in the French “practice of medicine.” Modern studies have shown that brushing with herbal toothpaste containing clove resulted in a significant reduction in salivary lactate dehydrogenase (LDH), improved cellular integrity, and reduced plaque and gingivitis (109). That hexane extract of *S. aromaticum* seeds exhibits preferential growth inhibitory activity against cariogenic pathogens in dental caries and may be used to treat dental caries (110). In addition, CE may also present new natural therapeutic potential by inhibiting dentin erosion (111).

### Nano technologies

In recent years, nanotechnology has rapidly evolved. A wide range of lipid nanostructures such as liposomes and solid lipid nanoparticles, metals, nanocrystals and polymeric particles have been tested in several drug delivery systems in different animal models. In addition, many nano-drug delivery systems containing *S. aromaticum* are being developed.

Metallic nanoparticles (NPs) have been widely used in cosmetics and medicine due to their unique antibacterial and antitumor properties. CE can be used as a reducing and stabilizing agent. CE-silver nanoparticles, synthesized by biosynthesis, showed good inhibitory activity against marine bacterial communities and *Nitzschia closterium* diatoms activity (112). Carboxymethyl cellulose structured silver-based nanocomposite (CMC-AgNPs) containing CE has shown antibacterial, *in vivo* anti-inflammatory, antileishmanial and antioxidant activities with low cytotoxicity (113). In addition, the synthesis of Au/Ag bimetallic nanoparticles by a single-step green route with CE significantly enhanced antioxidant and catalytic activity compared to individual monometallic nanoparticles (114).

Nano-encapsulation technology has been widely used in recent years. A study by Radün et al. (115) showed that the nano-encapsulated CEO had a strong antibacterial inhibitory capacity. CEO nanofibers formed by encapsulation in chitosan and polyethylene oxide polymers showed good antibacterial activity against *Staphylococcus aureus* and *Escherichia coli*, no cytotoxicity against humans fibroblast cell lines, and exhibited good wound healing potential (116). The retention of CEO in chitosan nanoparticles (ChNPs) was as high as 55.8-73.4%, and its antioxidant activity was significantly higher. Ashjazadeh et al. (117) also demonstrated that CE nanofibers showed the best granulation tissue by producing collagen and outperformed nanofibers such as nano zinc oxide in promoting wound healing in rats. Shetty et al. (118) found that ethosomal gel of the CEO was more effective in the treatment of cutaneous candidiasis than the pure CEO. In the croton oil-induced skin inflammation model in mice, it was found that nanofibers and nanoemulsions were more effective in the topical treatment of inflammation, and the efficacy of nanofibers was relatively higher than that of medicinal nanoemulsions (119). Plant essential oil is lipophilic, can easily cross cell membranes, and has greater anticancer efficacy potential. The CEO-based nanoemulsion system can increase drug retention time and improve bioavailability, making it a good candidate for cancer drug delivery systems. In addition, CEO nanoemulsions have greater potential in the food and agriculture industries. CEO-based nanoemulsions prepared by ultrasound using Tween 80 can be used as a natural delivery system to extend the shelf life of food products (120). The use of CEO-based nanoemulsions as green nanocarriers can significantly improve the solubility, bioavailability, and release of the pesticide Atrazine (ATZ). ATZ nanoemulsions also exhibited excellent herbicidal activity at low concentrations compared to commercial ATZ analogs (121).

### Food storage

*Syzygium aromaticum* showed beneficial advantages in antibacterial and antifungal activity, aromaticity and safety, especially against a wide range of food-borne microorganisms, making it a potential and valuable preservative in the food industry. The antifungal activity of clove is superior to that of lemongrass and thyme essential oils to prevent the natural preservation growth of fungi on dried apricots (122). Ju et al. (123) confirmed by accelerated storage tests that the CEO can extend the shelf life of food bakery products by 2–4 days under normal packaging. Hasheminejad et al. (124) found that encapsulation of CEO by ChNPs was more effective in extending the shelf life of food than CEO alone, and in a pomegranate shelf life and quality study, CEO-ChNPs improved the antifungal effect of CEO, effectively extending the shelf life by 54 days. Both CEO and CEO-based nanoemulsions inoculated into Talaga cheese were found to have significant inhibitory activity against foodborne pathogens such as *Listeria monocytogenes* and *Shigella flexneri*. Compared to CEO, CEO-based nanoemulsions significantly reduced the counts of inoculated pathogens from 8.2 to 1.5 log_10_cfu/g, showing greater antimicrobial effect (125). In addition, the addition of CEO to the structure of electrospun zein can better inhibit the growth of *Listeria monocytogenes* and *Escherichia coli*, effectively extending the shelf life of Iranian white cheese (126). The addition of clove powder to kimchi paste inhibited the growth of total aerobic and lactic acid bacteria, delayed the changes in O_2_ and CO_2_ concentrations and sugar and organic acid contents, and slowed down the decrease in pH, thus extending its shelf life (127). In addition, Eugenol-lean clove extracts can be used as a substitute for mustard flavoring in mayonnaise. They can improve its associated physical properties, giving it higher antioxidant activity and reducing capacity (128). Mixing the active ingredients in *S. aromaticum* into feeds has potential uses for improving meat quality, for example, adding *S. aromaticum* seeds to broiler chicken diets can significantly improve water retention, cooking loss percentage and tenderness of meat (129).

### Insecticidal efficacy

Plant-based biopesticides have been proposed to be the best pest control tools compared to conventional synthetic molecules. Many plants’ essential oils exhibit broad-spectrum insecticidal and repellent properties, are relatively non-toxic to mammals and fish and have gradually developed become potential alternatives to synthetic insecticides. *S. aromaticum* has been widely studied for its excellent insecticidal biological potential and higher safety for the environment and humans (130). The insecticidal potential of clove seed powder was evaluated by red palm weevil. It was found that *S. aromaticum* seeds powder at a dosage of 7 mg resulted in 100% mortality within three days (30). Terpenoids in *S. aromaticum* can reduce the respiratory rate of *Sitophilus granarius L.* exhibit a tropism effect, and prevent or delay the development of insecticide resistance (131). Viteri et al. (132) found that CEO had similar insecticidal activity to the synthetic pyrethroid insecticide deltamethrin, significantly affecting the oviposition of sublethally exposed *Callosobruchus maculatus* females and affecting their population growth. In addition, *S. aromaticum* showed high levels of repellency against fleas, aphids, nymphal instars, mites, mosquito species (Aedes aegypti, Anopheles gambiae, Culex quinquefasciatus etc.), termite and red imported fire ants etc. (133, 134). Toxicity assessment of clove powder, eugenol, eugenol acetate, and β-caryophyllene against red imported fire ants Solenopsis invicta Buren revealed that application of clove powder at 3 and 12 mg/cm^2^ provided 100% ant mortality within 6 h and repelled 99% within 3 h. Compared to eugenol acetate, β-caryophyllene and CEO, eugenol was the compound with the fastest action against red imported fire ants. And with increasing application rates, the LT_50_ values of the chemical compounds inclined exponentially (135). The findings suggest that *S. aromaticum* has potential as a natural source of insecticides.

### Other applications

While acetaldehyde is the main cytotoxin formed by alcohol metabolism and causes liver damage, extracellular matrix changes, inflammation, and hangover in heavy drinkers, clove polyphenol extract can accelerate the elimination of acetaldehyde from human blood, reduces hangover, and alleviates alcohol-related side effects (136). Clove has also been used worldwide as an anesthetic for various fish species, including several Amazon fish, Far Eastern catfish, small-sized tropical fish, and Pacific hagfish. Due to its better antioxidant effects, clove has also been used in recent years in studies on the role of skin barrier repair, where it activates the nuclear erythroid 2-related factor/antioxidant response element (Nrf2/ARE) signaling pathway to increase antioxidant activity. It significantly increases type I procollagen and elastin levels through TGF/Smad signaling and effectively ameliorates UVB-induced photoaging (137).

## Conclusion and prospect

This paper review the nutritional composition, phytochemistry, pharmacological effects and application prospects of *S. aromaticum* by combining traditional literature with modern evidence. Eugenol is the main phytoconstituent of *S. aromaticum*, which is involved in almost all the pharmacological activities of *S. aromaticum* and has been extensively studied in recent years. *S. aromaticum* also has a variety of nutritional and other phytoconstituents, such as β-Caryophyllene, α-Humulene, sesquiterpene, flavonoids, etc. These compounds are responsible for the powerful antioxidant and antibacterial properties of *S. aromaticum*. Respectively, no in-depth studies were carried out on individual substances to explore their pharmacological effects and mechanisms.

The current research on *S. aromaticum* mainly focuses on the active ingredients of flower buds (clove). A large amount of literature has proved that the CE has various pharmacological activities, such as antioxidant, antibacterial, anti-inflammatory, antiviral, analgesic, neuroprotective, hypoglycemic, anticancer, etc. Clove has been widely used in food, medicine, and cosmetics. The other parts of *S. aromaticum* (seeds, leaves, etc.) also have similar active ingredients as those in flower buds. However, no extensive studies were carried out on other parts of *S. aromaticum*. Thus, more research on other parts of the *S. aromaticum* and the compounds present is worth it.

In addition, although there is a large amount of pharmacological evidence in the literature that can lay the foundation for clinical studies on *S. aromaticum*, a search through the ClinicalTrials.gov and Chinese Clinical Trials Registry shows that there are still less than 30 registered clinical studies on *S. aromaticum*. These studies mainly focus on the effects of clove oil on pain and dental caries, as well as the clinical efficacy observation of Chinese medicine such as Dingxiang Shidi Decoction, Dingxiang Kaiwei Paste combined with other therapies. The results of the only clinical studies on *S. aromaticum* have proved that its biological activity is consistent with the results of preclinical studies. However, due to the small number and scope of clinical studies, it still cannot fully reflect the real clinical situation, and the clinical value of *S. aromaticum* has not been fully explored. Therefore, it is recommended that controlled clinical studies be conducted to understand more about the pharmacology, molecular mechanisms, and safety of *S. aromaticum*. In-depth studies on the bioactivity of the constituents, their structure-activity relationships and potential interactions (either synergistic or antagonistic) should be carried out.

In conclusion, *S. aromaticum* is an edible plant with various bioactive components and biological activities. It has a lot of opportunities for further development to improve its value and use in the food and pharmaceutical industries.

## Author contributions

XL coordinated technical support and funding. PG performed data collection and coordinated technical support. QX and ZX performed the study and drafted the manuscript. SW and ZC revised the manuscript. All authors read and approved the final manuscript.

## Abbreviations

CEO: clove essential oil
CE: clove extract
ECE: clove ethanol extract
ACE: the aqueous extract of clove
ChNPs: chitosan nanoparticles.
