# Supplementary material for: Recent advances in nutritional composition, phytochemistry, bioactive, and potential applications of Syzygium aromaticum L. (Myrtaceae)
Source: Front Nutr. 2022 Oct 14;9:1002147. doi: 10.3389/fnut.2022.1002147 (PMC9614275; doi:10.3389/fnut.2022.1002147)
Supplement: Supplementary file 1 [file Table_1.pdf]

Table S1. Volatile composition of different parts of *S. aromaticum*.

Classes	Chemical compound	Molecular formula	Extraction solvent	Biological activity	Reference
		Flower buds			
Aromatic	Eugenol	$C_{10}H_{12}O_2$	Water, Ethyl acetate, Diethyl ether, Methanol, n-Hexane	Antioxidant, Antibacterial, Antifungal, Antiviral, Antitumor, Anaesthetic, Insecticidal	(9), (21), (22), (23-27)
	Eugenyl acetate	$C_{12}H_{14}O_3$	Water, Ethyl acetate, Diethyl ether, Methanol, n-Hexane	Antioxidant, Antibacterial, Antifungal, Antiviral, Antitumor, Anaesthetic, Insecticidal	(9), (21), (24), (26), (27)
	Isoeugenyl acetate	$C_{12}H_{14}O_3$	Water	Antioxidant, Antibacterial	(22)
	Menthyl benzoate	$C_{17}H_{23}O_2$	Water	Antibacterial, Antifungal, Antiviral, Antioxidant, Antitumor, Anaesthetic, Insecticidal	(21)
	Ethyl benzoate	$C_9H_{10}O_2$	Water	Antibacterial, Antifungal, Antiviral, Antioxidant, Antitumor, Anaesthetic, Insecticidal	(21)
	Benzyl acetate	$C_9H_{10}O_2$	Water	Antibacterial, Antifungal, Antiviral, Antioxidant, Antitumor, Anaesthetic, Insecticidal	(21), (27)
	2-Phenyiethyl acetate	$C_{11}H_{13}ClO_2$	Water	Antibacterial, Antifungal, Antiviral, Antioxidant, Antitumor, Anaesthetic, Insecticidal	(21)
	(E)-Anethole	$C_{10}H_{12}O$	Water	Antibacterial, Antifungal, Antiviral, Antioxidant, Antitumor, Anaesthetic, Insecticidal	(21)
	Benzyl alcohol	C_7H_8O	Water	Antibacterial, Antifungal, Antiviral, Antioxidant, Antitumor, Anaesthetic, Insecticidal, Anti-type 2 diabetes	(21), (28)
	Menthyl eugenol	$C_{19}H_{25}O_2$	Water	Antibacterial, Antifungal, Antiviral,	(21), (27)

	Cinnamic aldehyde	C_9H_8O	Water	Antioxidant, Antitumor, Anaesthetic, Insecticidal Antibacterial, Antifungal, Antiviral, Antioxidant, Antitumor, Anaesthetic, Insecticidal	(21), (27)
	Ethyl cinnamate	$C_{11}H_{12}O_2$	Water	Antibacterial, Antifungal, Antiviral, Antioxidant, Antitumor, Anaesthetic, Insecticidal	(21)
	Benzyl tiglate	$C_{12}H_{14}O_2$	Water	Antibacterial, Antifungal, Antiviral, Antioxidant, Antitumor, Anaesthetic, Insecticidal	(21)
	Phenol	C_6H_5OH	Water	Antioxidant, Antibacterial	(22)
	Mesitylene	C_9H_{12}	Water	Antioxidant	(9)
	Psi-cumene	C_9H_{12}	Water	Antioxidant	(9)
	Hemimellitene	C_9H_{12}	Water	Antioxidant	(9)
	p-Cymene	$C_{10}H_{14}$	Water	Antioxidant	(9)
	3-Ethyl-o-xylene	$C_{10}H_{14}$	Water	Antioxidant	(9)
	4-Ethyl-o-xylene	$C_{10}H_{14}$	Water	Antioxidant	(9)
	1,2,3,4- Tetramethylfulvene	$C_{10}H_{14}$	Water	Antioxidant	(9)
	Prehnitene	$C_{10}H_{14}$	Water	Antioxidant	(9)
	2’3’4’Trimethoxyacetophenon	$C_{11}H_{14}O_4$	Methanol	Antioxidant, Antibacterial, Insecticidal	(25)
	Phenol,2-methoxy-4-(2-propenyl)	$C_{11}H_{14}O$	Methanol	Antioxidant, Antibacterial, Insecticidal	(25)
	Chavicol	$C_9H_{10}O$	Methanol	Antioxidant, Antifungal	(24)
	1,2,3-Benzenetriol	$C_6H_6O_3$	Methanol	Antioxidant, Antibacterial	(25)
	Asarone	$C_{12}H_{16}O_3$	Methanol	Antioxidant, Antibacterial	(25)
	Benzeneacetamide,N-(aminocarbonyl)-4-hydroxy-3-methoxy	$C_{19}H_{29}NO_3$	Methanol	Antioxidant, Antibacterial	(25)
	Phenol,2-methoxy-4-(methoxymethyl)	$C_9H_{12}O_3$	Methanol	Antioxidant, Antibacterial	(25)
	Benzaldehyde,4-ethyl	$C_9H_{10}O$	Methanol	Antioxidant, Antibacterial	(25)
	Thymyl methyl ether	$C_{11}H_{16}O$	Water	Anti-type 2 diabetes	(28)
Sesquiterpenoids	Benzyl benzoate	$C_{14}H_{12}O_2$	-	Antitumor	(27)
	β-Caryophyllene	$C_{15}H_{24}$	Water, Ethyl acetate, Diethyl ether, Methanol, n-Hexane	Antioxidant, Antibacterial, Antifungal, Antiviral, Antioxidant, Antitumor, Anaesthetic, Insecticidal	(9), (21), (22), (23-27)
	Caryophyllene oxide	$C_{15}H_{24}O$	Water,	Antioxidant,	(9), (21),

		Ethyl acetate, Diethyl ether, Methanol, n-Hexane	Antibacterial, Antifungal, Antiviral, Antitumor, Anaesthetic, Insecticidal	(22), (23-25), (27)
α-Cubebene	$C_{15}H_{24}$	Water, Methanolic	Antibacterial, Antifungal, Antiviral, Antioxidant, Antitumor, Anaesthetic, Insecticidal	(21), (23), (24), (27)
Copaene	$C_{15}H_{24}$	Water	Antibacterial, Antifungal, Antiviral, Antioxidant, Antitumor, Anaesthetic, Insecticidal	(21), (27)
α-Humulene	$C_{15}H_{24}$	Water, Ethyl acetate, Diethyl ether, Methanol, n-Hexane	Antioxidant, Antibacterial, Antifungal, Antiviral, Antitumor, Anaesthetic, Insecticidal	(9), (21), (22), (23), (24), (27)
α-Amorphene	$C_{15}H_{24}$	Water	Antibacterial, Antifungal, Antiviral, Antioxidant, Antitumor, Anaesthetic, Insecticidal	(21)
α-Muurolene	$C_{15}H_{24}$	Water, Methanol	Antibacterial, Antifungal, Antiviral, Antioxidant, Antitumor, Anaesthetic, Insecticidal	(21), (25)
γ-Cadinene	$C_{15}H_{24}$	Water, Petroleum ether	Antibacterial, Antifungal, Antiviral, Antioxidant, Antitumor, Anaesthetic, Insecticidal	(21), (22), (27)
β-Cadinene	$C_{15}H_{24}$	Methanol	Antifungal	(24)
Calamenene	$C_{15}H_{22}$	Water	Antibacterial, Antifungal, Antiviral, Antioxidant, Antitumor, Anaesthetic, Insecticidal	(21)
Calacorene	$C_{15}H_{20}$	Water	Antibacterial, Antifungal, Antiviral, Antioxidant, Antitumor, Anaesthetic, Insecticidal	(21)
Humulene oxide	$C_{15}H_{24}O$	Water	Antibacterial, Antifungal, Antiviral, Antioxidant,	(21), (27)

				Antitumor, Anaesthetic, Insecticidal	
	Humulenol	$C_{15}H_{24}O$	Water	Antibacterial, Antifungal, Antiviral, Antioxidant, Antitumor, Anaesthetic, Insecticidal	(21)
	Isolongifolene	$C_{15}H_{24}$	Water	Antioxidant	(9)
	(E,E)-Alpha-farnesene	$C_{15}H_{24}$	Methanol	Antifungal, Antioxidant, Antibacterial, Insecticidal	(24), (25)
	β-Guaiene	$C_{15}H_{24}$	-	Antitumor	(27)
	β-Elemene	$C_{15}H_{24}$	-	Antitumor	(27)
	β-Gurjunene	$C_{15}H_{24}$	-	Antitumor	(27)
	Farnesol acetate	$C_{17}H_{28}O_2$	Methanol	Antioxidant, Antibacterial	(25)
	1,4,7-Cycloundecatriene	$C_{15}H_{24}$	Methanol	Antioxidant, Antibacterial	(25)
Monoterpene	Linalool	$C_{10}H_{18}O$	Water	Antibacterial, Antifungal, Antiviral, Antioxidant, Antitumor, Anaesthetic, Insecticidal, Anti-type 2 diabetes	(21), (27), (28)
	α-Terpinyl acetate	$C_{12}H_{20}O_2$	Water	Antibacterial, Antifungal, Antiviral, Antioxidant, Antitumor, Anaesthetic, Insecticidal	(21), (27)
	Limonene	$C_{10}H_{16}$	Water	Antioxidant, Anti-type 2 diabetes	(28)
	Carvone	$C_{10}H_{14}O$	Water, Ethyl acetate	Antibacterial, Antifungal, Antiviral, Antioxidant, Antitumor, Anaesthetic, Insecticidal	(21), (24), (27)
	Terpinen-4-ol	$C_{10}H_{18}O$	Water	Anti-type 2 diabetes	(28)
	Camphene	$C_{10}H_{16}$	Water	Anti-type 2 diabetes	(28)
	α-Pinene	$C_{10}H_{16}$	Water	Anti-type 2 diabetes	(28)
	β-Pinene	$C_{10}H_{16}$	Water	Anti-type 2 diabetes	(28)
	Cis-ocimene	$C_{10}H_{16}$	Water	Anti-type 2 diabetes	(28)
	Myrcene	$C_{10}H_{16}$	Water	Anti-type 2 diabetes	(28)
	Pinene-2-ol	$C_{10}H_{16}$	Water	Anti-type 2 diabetes	(28)
	α-Thujene	$C_{10}H_{16}$	Water	Anti-type 2 diabetes	(28)
	Gamma terpinene	$C_{10}H_{16}$	Water	Anti-type 2 diabetes	(28)
	Neral	$C_{10}H_{16}O$	Water	Anti-type 2	(28)

				diabetes	
	Geranial	$C_{10}H_{16}O$	Water	Anti-type 2 diabetes	(28)
	Isoartemisia	$C_{10}H_{16}O$	Water	Anti-type 2 diabetes	(28)
	1,8-Cineole	$C_{10}H_{18}O$	Water	Antioxidant, Anti-type 2 diabetes	(28)
	Borneol	$C_{10}H_{18}O$	Water	Anti-type 2 diabetes	(28)
Diterpenes	Menthyl chavicol	$C_{19}H_{28}O$	Water	Antibacterial, Antifungal, Antiviral, Antioxidant, Antitumor, Anaesthetic, Insecticidal	(21), (27)
Alkanes	α-Copane	$C_{15}H_{26}$	Water	Anti-type 2 diabetes	(28)
Ketone	2-Heptanone	$C_7H_{14}O$	Water	Antibacterial, Antifungal, Antiviral, Antioxidant, Antitumor, Anaesthetic, Insecticidal	(21)
	2-Nonanone	$C_9H_{18}O$	Water	Antibacterial, Antifungal, Antiviral, Antioxidant, Antitumor, Anaesthetic, Insecticidal	(21), (27)
	2-Undecanone	$C_{11}H_{22}O$	Water	Antibacterial, Antifungal, Antiviral, Antioxidant, Antitumor, Anaesthetic, Insecticidal	(21)
	Musk ketone	$C_{14}H_{18}N_2O_5$	Methanol	Antifungal	(24)
Alcohol	2-Nonanol	$C_9H_{20}O$	Water	Antibacterial, Antifungal, Antiviral, Antioxidant, Antitumor, Anaesthetic, Insecticidal	(21)
	2-Heptanol	$C_7H_{16}O$	Water	Antibacterial, Antifungal, Antiviral, Antioxidant, Antitumor, Anaesthetic, Insecticidal	(21)
Esters	Menthyl octanoate	C9H18O2	Water	Antibacterial, Antifungal, Antiviral, Antioxidant, Antitumor, Anaesthetic, Insecticidal	(21)
	Ethyl hexanoate	$C_8H_{16}O_2$	Water	Antibacterial, Antifungal, Antiviral, Antioxidant, Antitumor,	(21)

	Ethyl octanoate	$C_{10}H_{20}O_2$	Water	Anaesthetic, Insecticidal Antibacterial, Antifungal, Antiviral, Antioxidant, Antitumor, Anaesthetic, Insecticidal	(21)
Acids	2,4,4-Trimethyl-3-(3-methylbutyl) cyclohex-2-enone	$C_{14}H_{24}O$	Methanol	Antioxidant, Antibacterial	(25)
	n-Hexadecanoic acid	$C_{16}H_{32}O_2$	Methanol	Antioxidant, Antibacterial	(25)
	9,12-Octadecadienoic acid(z,z)	$C_{18}H_{32}O_2$	Methanol	Antioxidant, Antibacterial	(25)
	Octadecanoic acid	$C_{18}H_{32}O_2$	Methanol	Antioxidant, Antibacterial	(25)
Others	4H-Pyran-4-one,2,3-dichloro-3,5-dihydroxyl-6-methyl	$C_9H_{15}C_{16}O_4P$	Methanol	Antioxidant, Antibacterial	(25)
			Flower		
Aromatic					
	Eugenol	$C_{10}H_{12}O_2$	Water	Antioxidant, Antibacterial, Antifungal, Antiviral, Antitumor, Anaesthetic, Insecticidal	(9)
	Eugenyl acetate	$C_{12}H_{14}O_3$	Water	Antioxidant, Antibacterial, Antifungal, Antiviral, Antitumor, Anaesthetic, Insecticidal	(9)
	Psi-cumene	C_9H_{12}	Water	Antioxidant	(9)
	Hemimellitene	C_9H_{12}	Water	Antioxidant	(9)
	2-Indanol	$C_9H_{10}O$	Water	Antioxidant	(9)
	p-Cymene	$C_{10}H_{14}$	Water	Antioxidant	(9)
	3-Ethyl-o-xylene	$C_{10}H_{14}$	Water	Antioxidant	(9)
	4-Ethyl-o-xylene	$C_{10}H_{14}$	Water	Antioxidant	(9)
	1,2,3,4- Tetramethylfulvene	$C_{10}H_{14}$	Water	Antioxidant	(9)
	Prehnitene	$C_{10}H_{14}$	Water	Antioxidant	(9)
Sesquiterpenoids					
	β-Caryophyllene	$C_{15}H_{24}$	Water	Antioxidant, Antibacterial, Antifungal, Antiviral, Antioxidant, Antitumor, Anaesthetic, Insecticidal	(9)
	Caryophyllene oxide	$C_{15}H_{24}O$	Water	Antioxidant, Antibacterial, Antifungal, Antiviral, Antitumor, Anaesthetic, Insecticidal	(9)
	α-Humulene	$C_{15}H_{24}$	Water	Antioxidant, Antibacterial, Antifungal, Antiviral, Antitumor, Anaesthetic, Insecticidal	(9)
			Leaves		
Aromatic	Eugenol	$C_{10}H_{12}O_2$	Water	Antioxidant,	(29)

	Methyleugenol	$C_{11}H_{14}O_2$	Water	Antibacterial, Antifungal, Antiviral, Antitumor, Anaesthetic, Insecticidal antioxidant	(29)
	Isoeugenol	$C_{10}H_{12}O_2$	Water	antioxidant	(29)
	Methyl isoeugenol	$C_{11}H_{14}O_2$	Water	antioxidant	(29)
	Eugenyl acetate	$C_{12}H_{14}O_3$	Water	Antioxidant, Antibacterial, Antifungal, Antiviral, Antitumor, Anaesthetic, Insecticidal	(29)
	Isoeugenyl acetate	$C_{12}H_{14}O_3$	Water	Antioxidant, Antibacteria	(29)
	Methyl salicylate	$C_8H_8O_3$	Water	antioxidant	(29)
	Chavicol	$C_9H_{10}O$	Water	Antioxidant, Antifungal	(29)
Sesquiterpenoids	β-Caryophyllene	$C_{15}H_{24}$	Water	Antioxidant, Antibacterial, Antifungal, Antiviral, Antioxidant, Antitumor, Anaesthetic, Insecticidal	(29)
	Caryophyllene oxide	$C_{15}H_{24}O$	Water	Antioxidant, Antibacterial, Antifungal, Antiviral, Antitumor, Anaesthetic, Insecticidal	(29)
	α-Humulene	$C_{15}H_{24}$	Water	Antioxidant, Antibacterial, Antifungal, Antiviral, Antitumor, Anaesthetic, Insecticidal	(29)
	Humulene oxide	$C_{15}H_{24}O$	Water	Antibacterial, Antifungal, Antiviral, Antioxidant, Antitumor, Anaesthetic, Insecticidal	(29)
	α-Clovene	$C_{15}H_{24}$	Water	antioxidant	(29)
	(E,E)-Alpha-farnesene	$C_{15}H_{24}$	Water	Antifungal, Antioxidant, Antibacterial, Insecticidal	(29)
Monoterpene	Limonene	$C_{10}H_{16}$	Water	Antioxidant, Anti-type 2 diabetes	(29)
Diterpenes	Menthyl chavicol	$C_{19}H_{28}O$	Water	Antibacterial, Antifungal, Antiviral, Antioxidant, Antitumor, Anaesthetic, Insecticidal	(29)
Alkanes	1,8-Cineole	$C_{10}H_{18}O$	Water	Antioxidant, Anti-type 2	(29)

				diabetes	
Alcohol	Caryophyllene alcohol	$C_{15}H_{26}O$	Water	antioxidant	(29)
Ethers	Limonene oxide	$C_{10}H_{16}O$	Water	antioxidant	(29)
		Seeds			
Aromatic					
	Eugenol	$C_{10}H_{12}O_2$	Petroleum ether	Antioxidant, Antibacterial, Antifungal, Antiviral, Antitumor, Anaesthetic, Insecticidal	(30)
	Acetyl eugenol	$C_{12}H_{14}O_3$	Chloroform	Insecticidal	(30)
	Eugenyl acetate	$C_{12}H_{14}O_3$	Chloroform	Antioxidant, Antibacterial, Antifungal, Antiviral, Antitumor, Anaesthetic, Insecticidal	(30)
	3-Allyl guaiacol	$C_{10}H_{12}O_2$	Chloroform	Insecticidal	(30)
	1-Naphthalenol	$C_{10}H_8O$	Chloroform	Insecticidal	(30)
	2’3’4’Trimethoxyacetophenon	$C_{11}H_{14}O_4$	Chloroform, Petroleum ether	Antioxidant, Antibacterial, Insecticidal	(30)
	Benzene	C_6H_6	Petroleum ether	Insecticidal	(30)
	Phenol,2-methoxy-4-(2-propenyl)	$C_{11}H_{14}O$	Petroleum ether	Antioxidant, Antibacterial, Insecticidal	(30)
Sesquiterpenoids					
	β-Caryophyllene	$C_{15}H_{24}$	Chloroform	Antioxidant, Antibacterial, Antifungal, Antiviral, Antioxidant, Antitumor, Anaesthetic, Insecticidal	(30)
	Caryophyllene oxide	$C_{15}H_{24}O$	Petroleum ether	Antioxidant, Antibacterial, Antifungal, Antiviral, Antitumor, Anaesthetic, Insecticidal	(30)
	Copaene	$C_{15}H_{24}$	Chloroform, Petrolium ether	Antibacterial, Antifungal, Antiviral, Antioxidant, Antitumor, Anaesthetic, Insecticidal	(30)
	α-Humulene	$C_{15}H_{24}$	Chloroform, Petrolium ether	Antioxidant, Antibacterial, Antifungal, Antiviral, Antitumor, Anaesthetic, Insecticidal	(30)
	γ-Cadinene	$C_{15}H_{24}$	Petroleum ether	Antibacterial, Antifungal, Antiviral, Antioxidant, Antitumor, Anaesthetic, Insecticida	(30)
	Humulene oxide	$C_{15}H_{24}O$	Chloroform	Antibacterial, Antifungal,	(30)

				Antiviral, Antioxidant, Antitumor, Anaesthetic, Insecticidal	
	Germacrene-d	$C_{15}H_{24}$	Chloroform, Petroleum ether	Insecticidal	(30)
	(E,E)-Alpha-farnesene	$C_{15}H_{24}$	Chloroform, Petroleum ether	Antifungal, Antioxidant, Antibacterial, Insecticidal	(30)
	Cadina-1,4-diene	$C_{15}H_{24}$	Chloroform, Petroleum ether	Insecticidal	(30)
	Bicyclo [7.2.0] undec-4-ene	$C_{11}H_{18}$	Petroleum ether	Insecticidal	(30)
	Caryophylla-4(12),8(13)-dien-5	$C_{15}H_{22}O$	Petroleum ether	Insecticidal	(30)
Diterpenes	Menthyl chavicol	$C_{19}H_{28}O$	Chloroform, Petroleum ether	Antibacterial, Antifungal, Antiviral, Antioxidant, Antitumor, Anaesthetic, Insecticidal	(30)
Alkanes	1-Methylene-2b-hydroxymethyl-3-4B-(3-methylbut-2-enyl) cyclohexane	-	Chloroform	Insecticidal	(30)
	Cyclohexane	C_6H_{12}	Petroleum ether	Insecticidal	(30)
	Cycloheptane	C_7H_{14}	Petroleum ether	Insecticidal	(30)
	Heptane	C_7H_{16}	Petroleum ether	Insecticidal	(30)
	Nonane	C_9H_{20}	Petroleum ether	Insecticidal	(30)
	Tritetracontane	$C_{43}H_{88}$	Petroleum ether	Insecticidal	(30)
Alcohol	Cyclohexanol	$C_6H_{12}O$	Petroleum ether	Insecticidal	(30)
Esters	9-Methyl-10,12-hexadecadien-1-	$C_{19}H_{34}O_2$	Petroleum ether	Insecticidal	(30)

Note：“-” indicates that the item is not retrieved.
